# Development of microcatheter tube extrusion angle estimation system using convolutional neural network segmentation

**DOI:** 10.1038/s41598-023-45759-z

**Published:** 2023-10-27

**Authors:** Seung Hyun Jeong, Sang Heon Lee, Hong-In Won

**Affiliations:** 1 School of Mechatronics Engineering, Korea University of Technology and Education, Cheonan-si, 31253 Republic of Korea; 2 Manufacturing AI Research Center, Korea Institute of Industrial Technology, Cheonan-si, 31056 Republic of Korea

**Keywords:** Biomedical engineering, Mechanical engineering

## Abstract

This study presents a deep learning-based monitoring system for estimating extrusion angles in the manufacturing process of microcatheter tubes. Given the critical nature of these tubes, which are directly inserted into the human body, strict quality control is imperative. To mitigate potential quality variations stemming from operator actions, a system utilizing a convolutional neural network to precisely measure the extrusion angle—a parameter with profound implications for tube quality—is developed. Until now, there has been no method to estimate the extrusion angle of resin being extruded in real-time. In this study, for the first time, a method using deep learning to estimate the angle was proposed. This innovative system comprises two RGB cameras capturing both front and side perspectives. The acquired images undergo segmentation via a meticulously trained convolutional neural network. Subsequently, the extrusion angle is accurately estimated through the application of principal component analysis on the segmented image. The usefulness of the proposed system was rigorously confirmed through comprehensive validation measures, including mean intersection over union (mIoU), mean absolute angle error (MAE), and inference time, using a real-world dataset. The attained metrics, with an mIoU of 0.8848, MAE of 0.5968, and an inference time of 0.0546, unequivocally affirm the system’s suitability for enhancing the catheter tube extrusion process.

## Introduction

**Figure 1:**
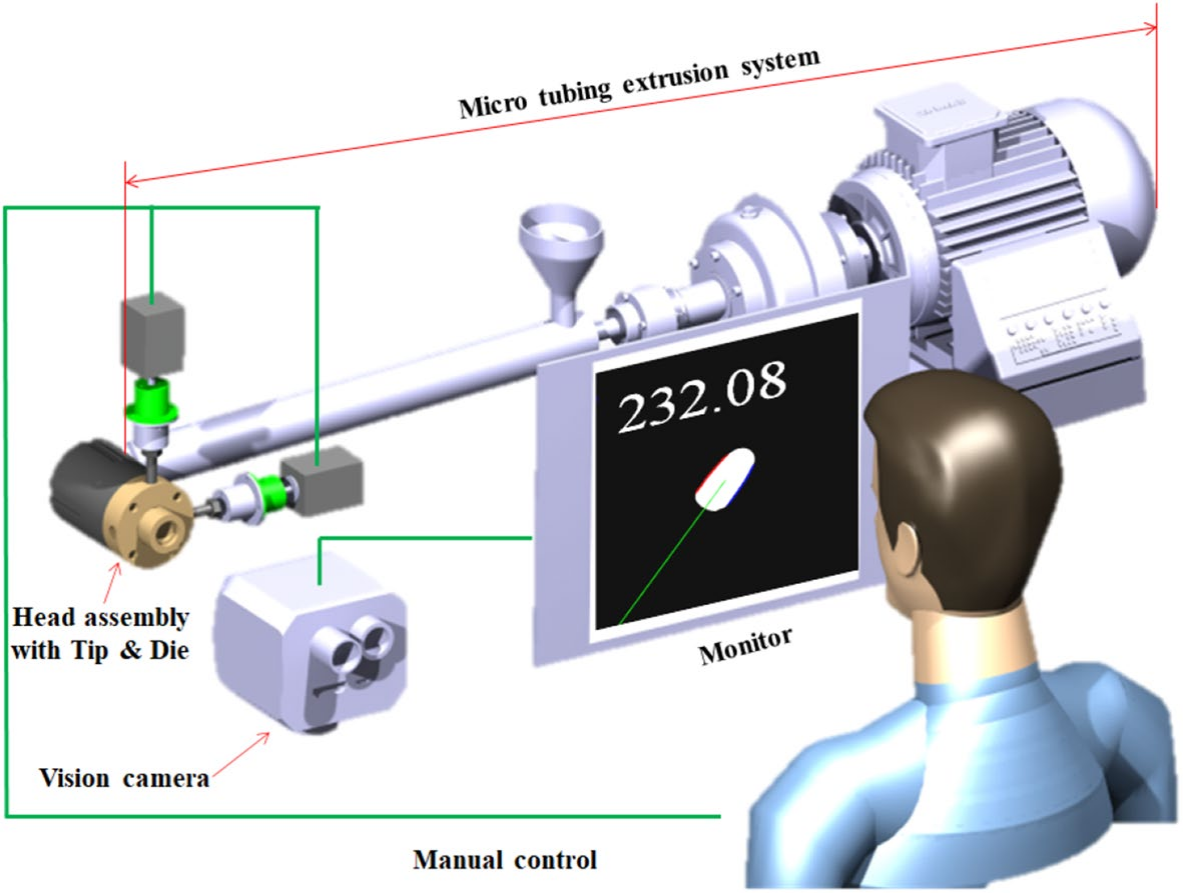
Conceptual diagram of microcatheter tube extrusion angle measurement system.

In this study, in order to improve the efficiency of the catheter tube extrusion process and minimize the waste of materials for the initial condition setting, research was conducted to quantify the extrusion angle of the tube based on deep learning. Due to the seriousness of environmental problems, green technology is becoming increasingly important. Especially in green technology, technologies that improve efficiency by minimizing defective products generated during the manufacturing process and shortening the process setting time required to obtain good products are very important^1–3^. In order to meet the needs of the era of green technology, research on artificial intelligence-based techniques for the extrusion process was conducted in this study. The extrusion process is a traditional method with a long history, and most of the facilities related to it are automated. However, even if most facilities are automated, the initial process setting for production had to be carried out through the operator. The extrusion process covered in this study is a process for manufacturing catheter tubes for coronary artery surgery^4^. As mentioned, this process equipment is mostly automated, but it had to rely on qualitative inspection by operators to set the initial process conditions for tube production. In the extrusion process dealt with in this study, the operator visually judged the extrusion angle of the tube and set the process conditions. Therefore, if the extrusion angle, which affects the quality of the tube, can be quantified during the process, the setting time can be shortened, and the waste of materials caused by trial and error can be reduced. For these reasons, an inspection system that quantifies the extrusion angle of the resin through deep learning during the tube extrusion process was developed, as shown in Fig. 1.

Extrusion is a tube manufacturing method in which a metal or polymeric material is stretched at a constant rate by applying thermal and compressive energy. The polymer extrusion method applied to the catheter tube melts solid polymer pellets and then molds them into a desired shape using a tip and a die. Due to the properties of a non-Newtonian fluid whose viscosity changes according to temperature and shear stress, it is very difficult to simply define the flow characteristics of molten polymer^5–7^. In addition, it is challenging to anticipate the final shape of the product due to production-related occurrences, like swelling and melt fracture. To obtain high-quality tubes, therefore, it is crucial to design and monitor the process effectively^8^.

With this in mind, deep learning is actively applied in various fields for green manufacturing. Deep learning algorithms, especially those that recognize images, such as classification^9^, detection^10–12^, and segmentation^13–15^, have shown accuracy surpassing human levels in certain areas due to significant advances. As a result of these remarkable advances, deep learning algorithms are being actively applied in monitoring manufacturing processes and inspecting products instead of human labor. In this regard, an U-net based segmentation algorithm was applied to detect smearing defects which are one of the main defects of screening printing^13^. This study segmented products from the roll-to-roll screening printing process and calculated the smearing area to detect defects. Also, a deep learning-based classification model was applied to the monitoring of directed energy deposition in additive manufacturing^16^. The study used thermal images to classify the condition of the process into 4 classes - normal, low powder, low speed, and high speed. Also, a failure known as a “spaghetti-shape error” in the material extrusion process was detected by using the Visual Geometry Group Network(VGGNet)^17^. In this research, the pre-trained model was tested on a 3D printer monitoring system to monitor whether a fault occurs. Moreover, a convolutional Long Short-Term Memory (LSTM) autoencoder was utilized to extract a significant quality measure from videos captured from a front-facing perspective during the bioprinting process^18^.

Although many studies have been conducted, there are few cases where deep learning is applied to the manufacturing process of medical tubes covered in this study. The developed algorithm is a method of quantifying the extrusion angle of resin based on deep learning. The proposed method uses deep learning-based segmentation to segment the resin and outer line and quantifies the extrusion angle using the pixel coordinates of the outer line segments. The developed technique can be used not only to determine whether there is an abnormality in the process, but also to be used as a judgement index for tightening or loosening the bolt that adjusts the relative position of the tip and die.

## Methods

### Microcatheter tube extrusion angle measurement system

**Figure 2:**
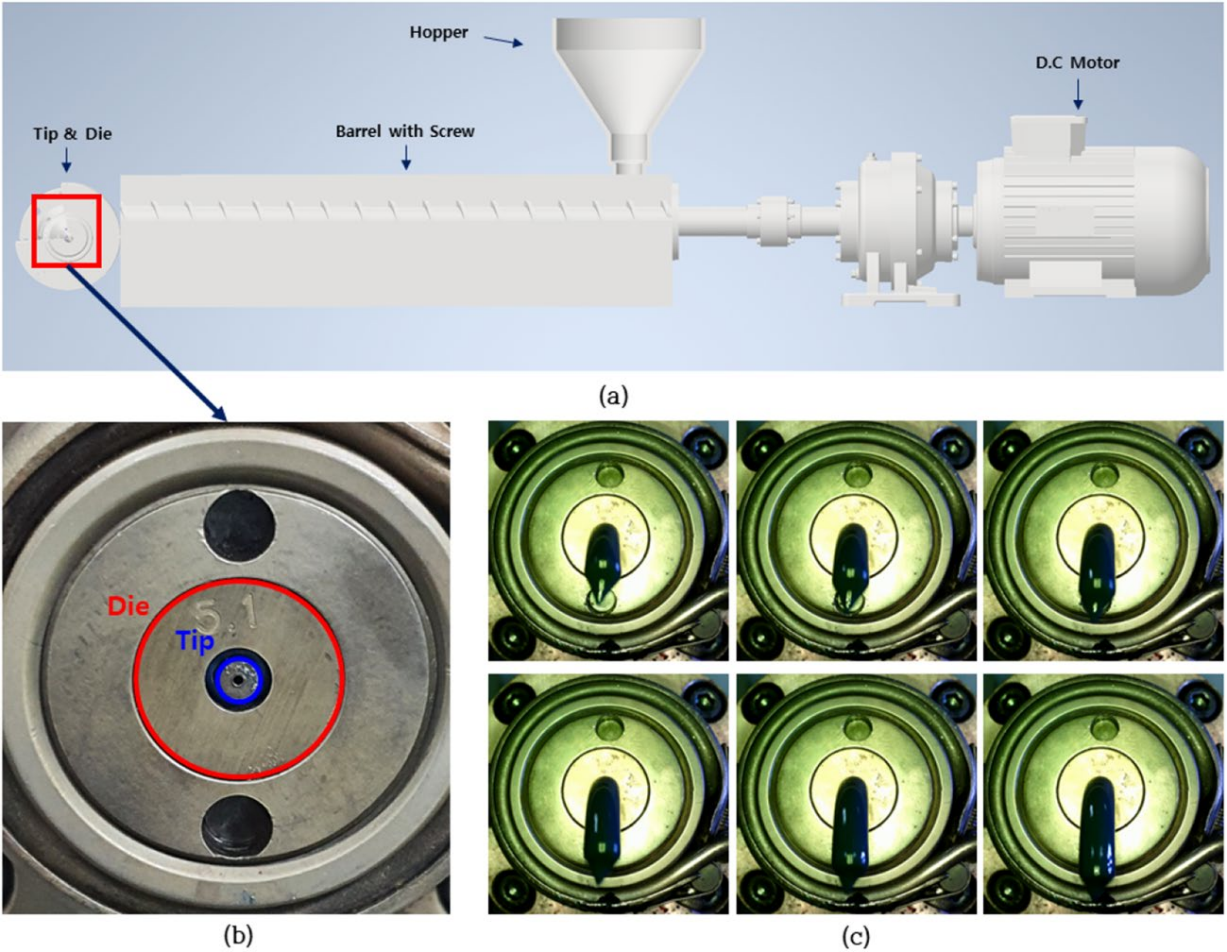
Structure of tube extrusion equipment and the front of the tube resin discharge part. (**a**) tube extrusion equipment, (**b**) die and tip of the extrusion, and (**c**) resin extrusion.

In the microcatheter extrusion process, a tube is manufactured by applying pressure to a metal or polymer material with thermal energy, elongating it at a constant speed, and then cooling it. The tube from the extrusion process becomes a part of a medical catheter. Fig. 2 shows the components of the extrusion process. In the polymer extrusion process, solid polymer pellets are introduced through a hopper and melted in a heating cylinder.

**Figure 3:**
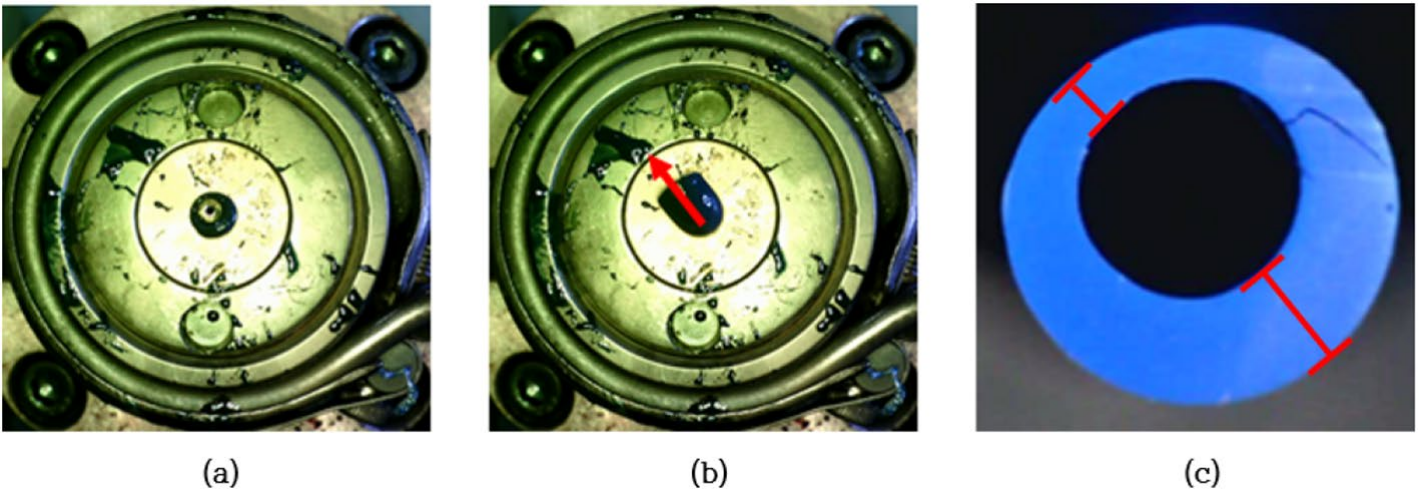
The effect of relative positions of die and tip. (**a**) An abnormal position of die and tip, (**b**) an inappropriate extrusion due to abnormal position, and (**c**) a thickness variation defect due to the abnormal position.

The melted resin passes through the equipment shown in Fig. 2a and is extruded through the tip shown in Fig. 2b. Figure 2c shows the images taken from the front of the extrusion part where the molten resin is ejected. In this extrusion process, numerous variables affect the quality of the tube. Among them, the relative positions of the die and the tip affect the amount of polymer extruded according to the position of the cross-section. For example, if the tip is moved and located on the left and top, as shown in Fig. 3a, the right and lower areas are relatively wider than other areas, a large amount of molten polymer is extruded on that side, which finally results in an extrusion deflected to the upper left, as shown in Fig. 3b. Due to this phenomenon, a cross-sectional shape having a non-uniform thickness is obtained as shown in Fig. 3c. Therefore, to date, in order to eliminate such defects of non-uniform cross-section, pre-production samples have been produced and the position of the die and tip have been manually fine-tuned through visual inspection until the biased extrusion state is eliminated.

Fiure 4 Depicts a manual fine-tuning process according to the operator’s qualitative judgement. As shown, the operator adjusts the extrusion angle by tightening or loosening the bolts located on the upper and right sides of the ejection part.

**Figure 4:**
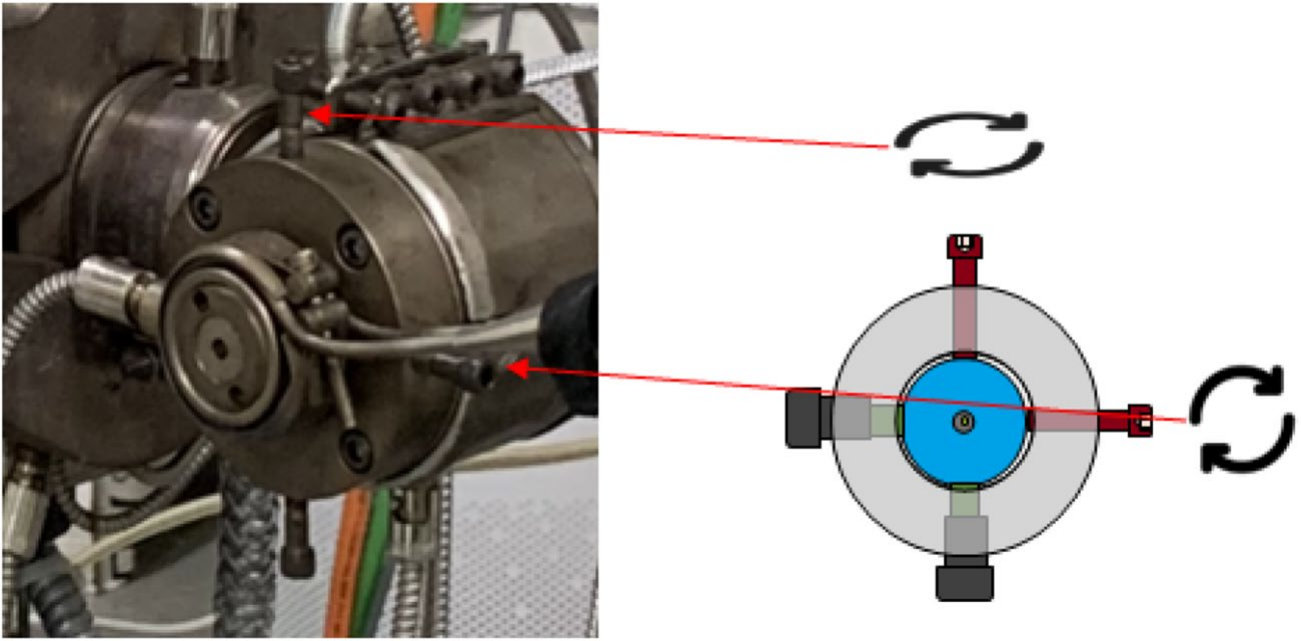
Manual control of the die position according to the direction of the extruded resin.

### The machine vision inspection system for extrusion angle inference

Unfortunately, the fine-tuning process mentioned in the previous section has a negative effect because the non-uniform quality of the tube can be obtained depending on the condition and experience of an operator. However, there is no existing standardized method for measuring extrusion angle. Therefore, the method to measure the extrusion angle in this study is proposed for the first time. In the process, data is acquired from a position where the discharge part is viewed from the front, and the inspector uses the acquired image to mark the left and right edge areas. The angle calculated by principal component analysis using the marked edge is considered the accurate extrusion angle. Therefore, if the deep learning algorithm accurately extracts the marked left and right edges, it will accurately predict the resin extrusion angle. In this study, a process diagnosis system based on machine vision inspection was developed to create a process that obtains tubes of uniform quality without depending on the condition and experience of the operator.

**Figure 5:**
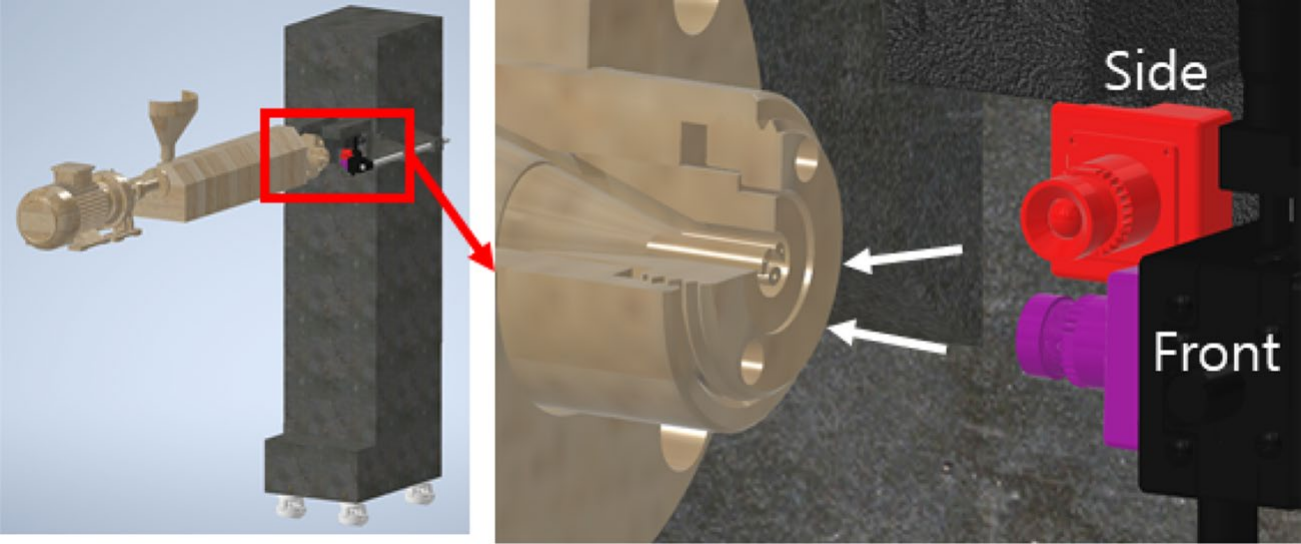
The manual control of the die position and visual inspection of the extruded resin.

In the vision-based measurement system, as shown in Fig. 5, two cameras are used to capture the front and side views of the tube extrusion.

**Figure 6:**
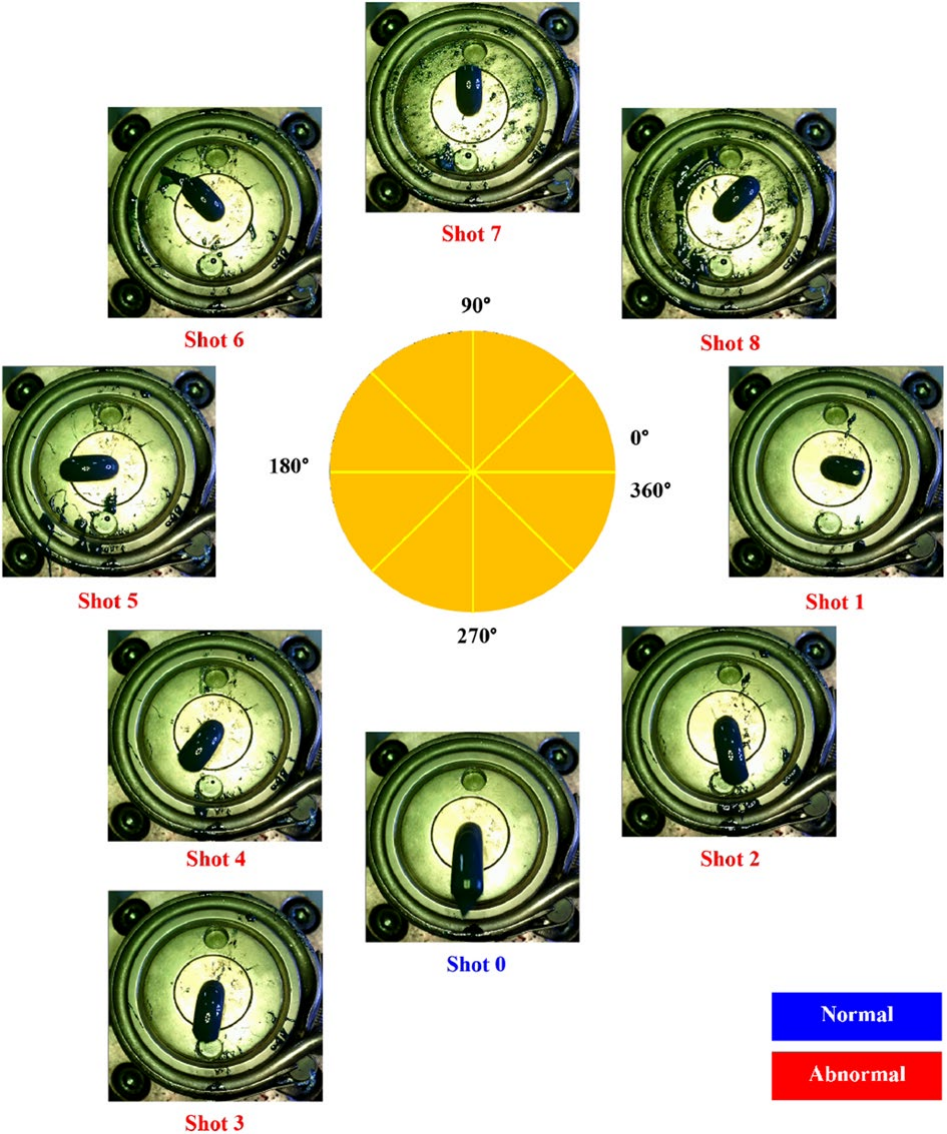
Frontal images taken while adjusting the relative position of the die and tip.

**Figure 7:**
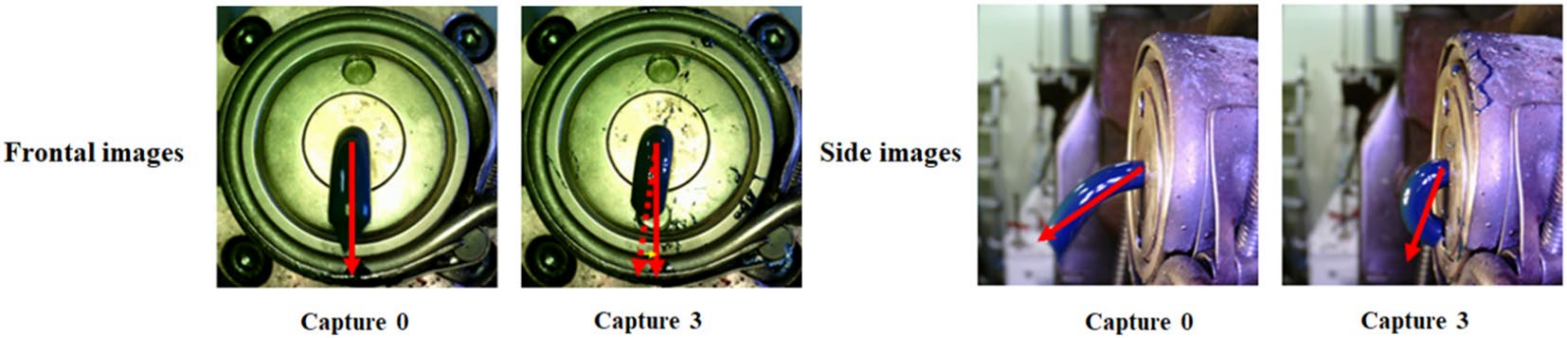
Side images taken at the relative positions for case 0 and case 3.

Using the system, the relative position of the die and tip was adjusted to identify the trend of the captured image. Figure 6 represents the frontal images captured by changing the relative position of the die and tip. A side camera was added to the system to deal with the situations not identified in the frontal image. It may be difficult to distinguish the extrusion resin direction whether the tip is located at the bottom or correct position because the extrusion angle of the tube is close to 270 degrees in both cases, as shown in Fig. 7.

**Figure 8:**
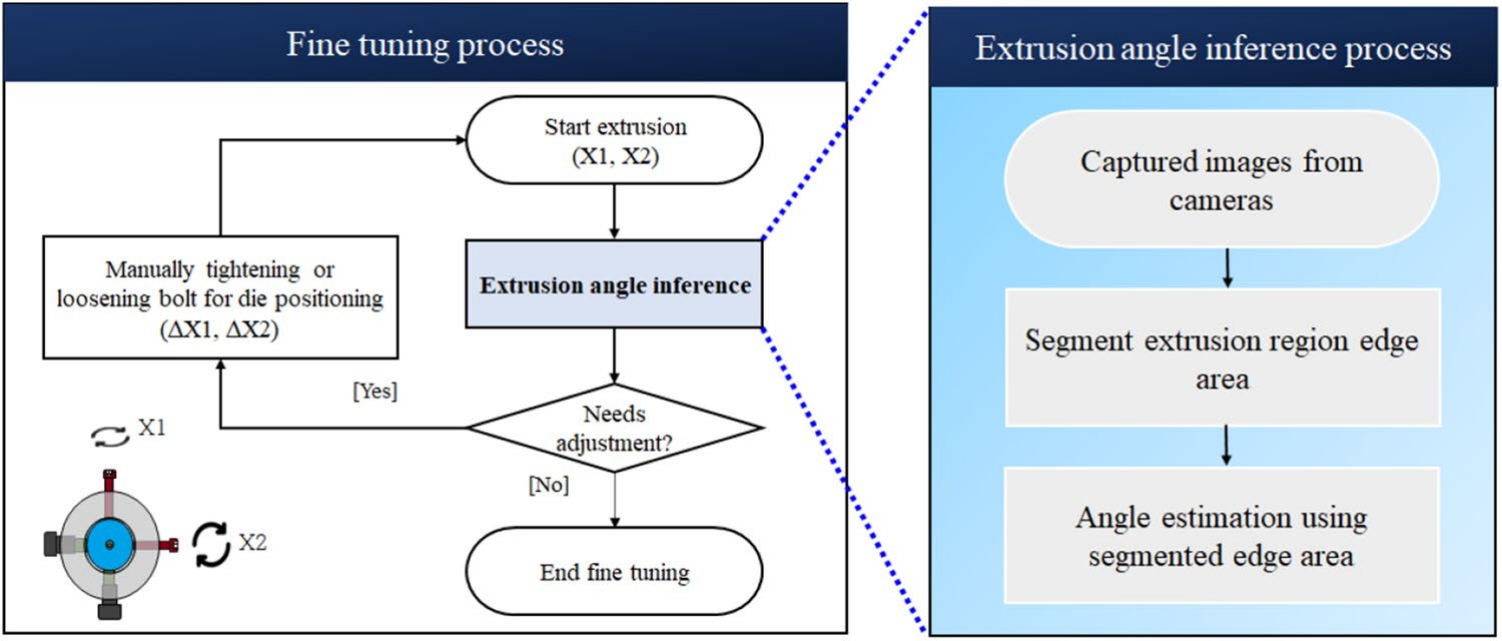
Fine tuning process using extrusion angle inference process.

Through the machine vision inspection system, the previous fine-tuning process was changed to a process using an algorithm that quantifies the extrusion angle, as shown in Fig. 8. The way to quantify the extrusion angle is to estimate the angle by using the segmented edge area after deep learning-based segmentation from the captured image, as shown in the blue box to the right of Fig. 8. Note that this study was limited to the extrusion angle inference system indicated by the blue box in Fig. 8. A detailed description of the angle estimation algorithm will be explained in the next subsection.

### Tube extrusion angle estimation algorithm

The tube extrusion angle estimation algorithm using the machine vision inspection system is explained in this section. The angle estimation algorithm is divided into two stages. In the first stage, the edge area of the resin is segmented by using deep learning-based segmentation in the captured image. In the second stage, the angle is estimated by performing principal component analysis on the pixel coordinates of the segmented edge area. Detailed descriptions of these two parts of the algorithm are given in the following subsection.

#### Resin edge segmentation

**Figure 9:**
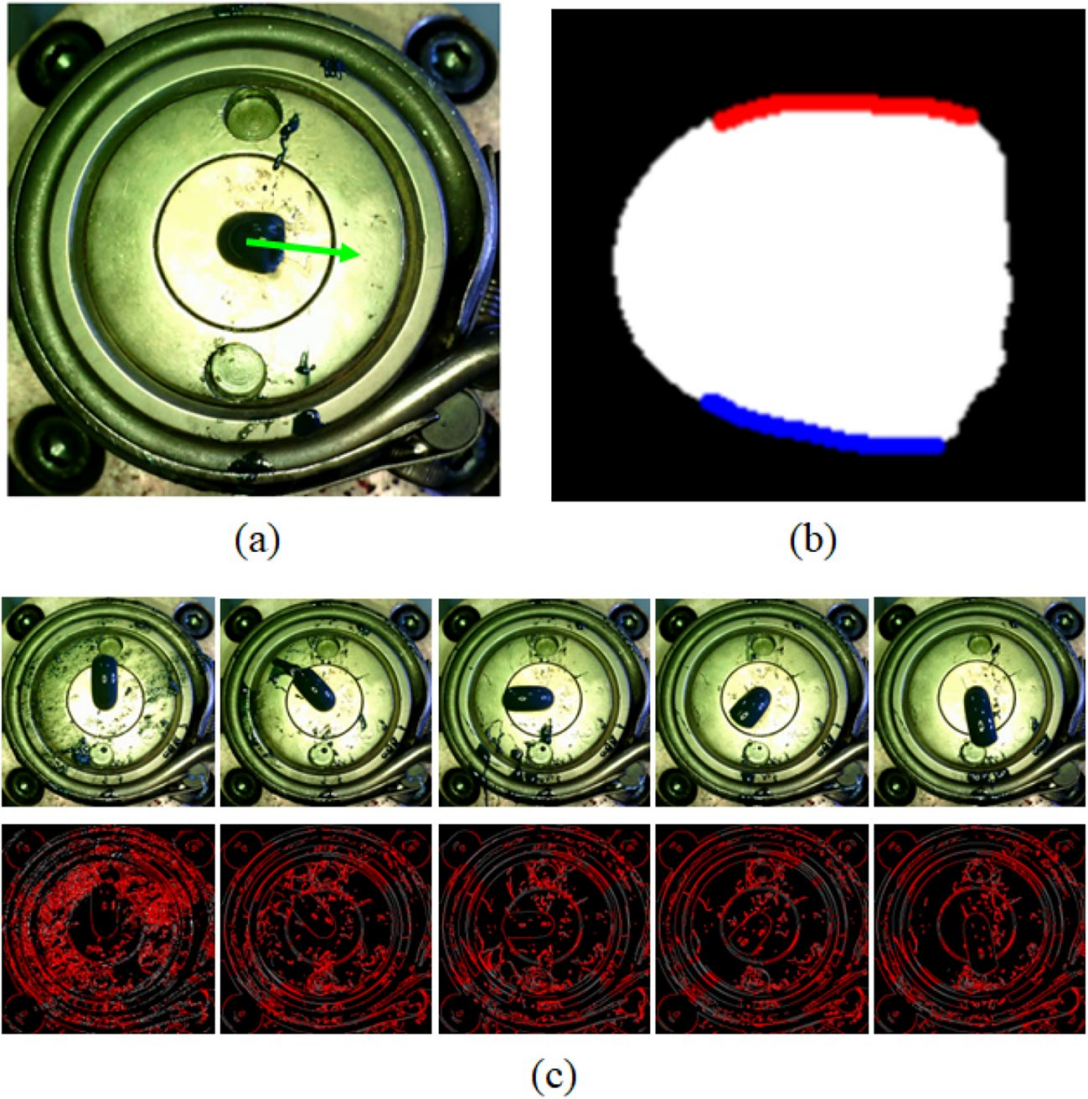
Difficulties arises when applying traditional image processing for resin edge detection. (**a**) the captured frontal image sample, (**b**) the edge that must be extracted from the image for the angle estimation, and (**c**) the result of line detection using hough transformation and Canny’s criteria to frontal images obtained in various situations. For image filtering, 5 by 5 gaussian filter was used. For binarization, Canny’s criteria was applied with minimum and maximum threshold 50 and 150, respectively. In an environment with contamination and changes in brightness, it is almost impossible to split only the edges of the resin.

In order to estimate the extrusion angle representing the ejection direction of resin, it is necessary to segment the edge area. Although there exists a line-detection algorithm based on the traditional hough transformation^19^ and Canny’s criteria^20^, it is very difficult to segment the edge for various cases because heuristic post-processing, which cannot be generalized, must be performed to find the start and end points of the edge area. Figure 9 expresses the difficulties that arise when applying traditional image processing. Figure 9 (a) is the frontal image, (b) is the edge that must be extracted from the image, and (c) is the result of line detection by applying hough transformation and Canny’s criteria to images obtained in various situations. For image filtering, 5 by 5 gaussian filter was used. For binarization, Canny’s criteria was applied with minimum and maximum threshold 50 and 150, respectively. As shown, due to the disturbance caused by contamination and brightness changes, etc., accurate and stable resin edge detection is almost impossible. Additionally, although this study dealt with one type of resin material and cross-sectional shape, there are numerous materials and cross-sectional shapes. If the deep learning model developed once, it can easily adapt to changes in resin material or cross-sectional shape, needing only the data to be updated and retrained. Therefore, this study adopts deep learning-based segmentation.

Therefore, a deep learning-based segmentation method is appropriate for robustly segmenting edge areas even under conditions where various disturbances exist. As shown in Fig. 9d, the captured images were labeled with a total of 4 classes, including the resin, the left and right sides of the edge line segment, and other areas. The extrusion angle of the resin is calculated by averaging the angles of each line segment in the left and right edge areas. Therefore, the left and right edge regions were separately labeled.

**Figure 10:**
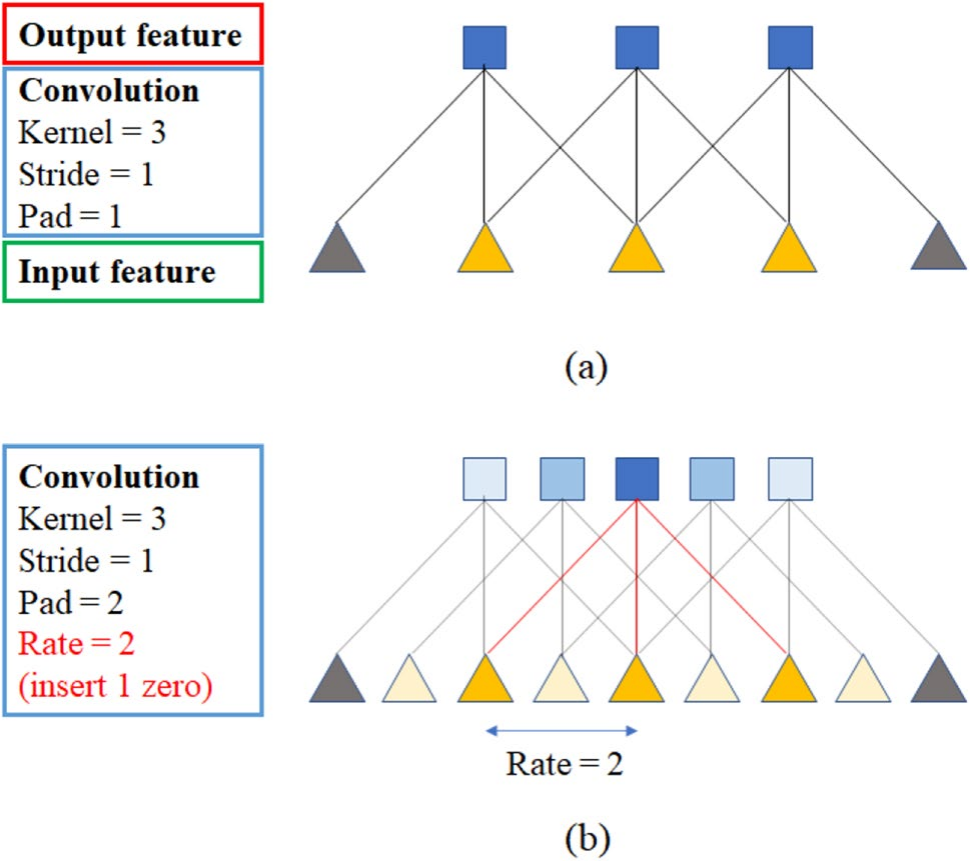
Description of 1-D atrous convolution. (**a**) Standard convolution for sparse feature extraction, and (**b**) atrous convolution with rate $r=2$ for dense feature extraction.

**Figure 11:**
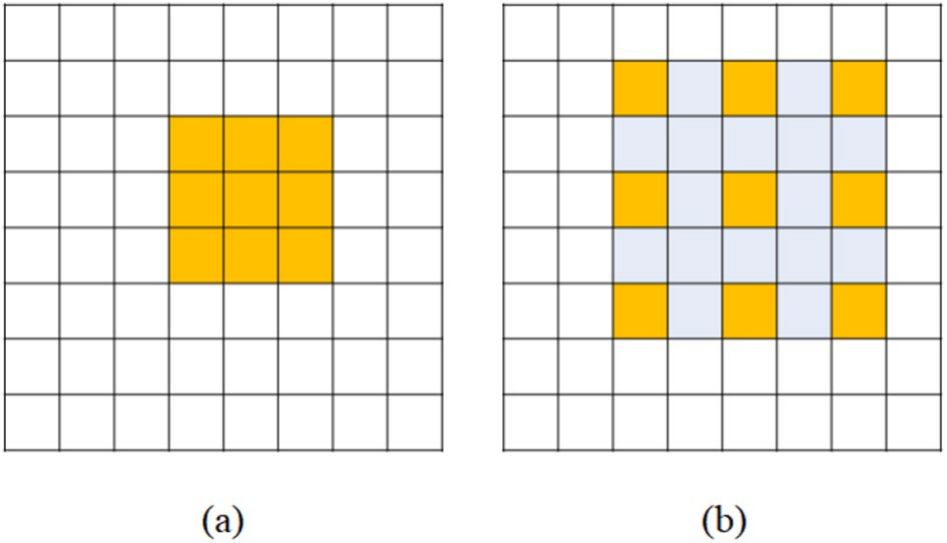
A description of 2-D atrous convolution. (**a**) An atrous convolution with $kernel=3$, $stride=1$, and $rate=1$, and (**b**) atrous convolution with $kernel=3$, $stride=1$, and $rate=2$.

For the edge segmentation, a model suitable for the characteristics of the acquired image must be used. As the edge area occupies a relatively small area, the feature may be lost in the feature extraction step of down-sampling. Therefore, it is necessary to use a model that can extract features of dense regions well. For this reason, a segmentation model based on an atrous convolution was used in this study^21,22^. In the one-dimensional atrous convolution with rate parameter *r*, the output *y*[*i*] of the input signal *x*[*i*] can be defined as follows.

1 $$\begin{aligned} y[i]=\sum _{K}^{k=1}x[i+r\cdot k]w[k], \end{aligned}$$

where *w*[*k*] is a filter with length *K*. As represented in Fig. 10, if the rate $r=1$, the atrous convolution is the same as a standard convolution. (write atrous convolution advantages) By using the described atrous convolution, the number of parameters calculated according to the kernel size given to the atrous convolution is the same as that of general convolution, but the receptive field area is extended by inserting a pixel value of 0 between kernels by the dilation rate to correspond to features of various sizes. For example, a 2-dimensional convolution with a kernel size of 3 accepts 9 parameters and 3 by 3 scale information, whereas an atrous convolution with a dilation rate of 2 and a kernel size 3 uses the same 9 parameters, but by inserting 0 as much as the dilation rate between the kernels, it is possible to secure the same acceptance area as the convolution with a kernel size of 5, as shown in Fig. 11. Thanks to the atrous convolution, the field of view of the filter can be extended to incorporate larger contexts with the same amount of computation.

**Figure 12:**
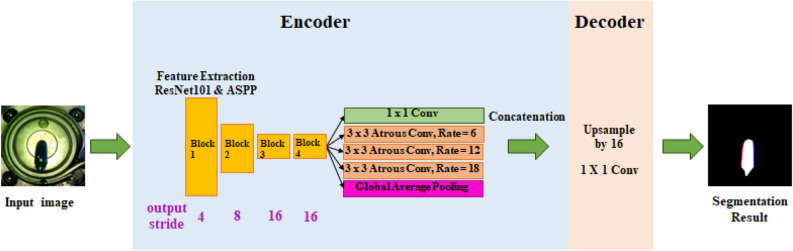
Segmentation network architecture using ResNet101 and ASPP for feature extraction.

By using the two-dimensional atrous convolution, atrous spatial pyramid pooling can be defined. The module of the atrous spatial pyramid pooling (ASPP) is added after the feature extractor, such as resnet and mobilenet, and is composed of two parts, ASPP and image-level features. The architecture of the segmentation network using Resnet 101 and ASPP as feature extractors is shown in Fig. 12. According to previous research^21^, There are three main advantages from a practical standpoint. The first advantage is speed. Due to atrous convolution, its receptive field area is extended with same number of weights and therefore inference speed can be accelerated without sacrificing accuracy. The second advantage is accuracy. The model uses ASPP achieved promising accuracies for various challenging datasets. The third advantage is simplicity. Because the Atrous convolution kernel stably implemented in a representative deep learning library, it is easy to apply.

For the training of the neural network, the output of the ASPP module is used to calculate the cross-entropy loss which is widely used in multi-class segmentation, which is calculated as follows.

2 $$\begin{aligned} L_{CE}=-\sum _{n}^{i=1}t_{i}log(p_{i}), \end{aligned}$$

where $t_i$ is the truth label and $p_i$ is the softmax probability for the *i*th class. Note that *n* is the total number of classes.

#### Extrusion angle estimation using the segmented image

**Figure 13:**
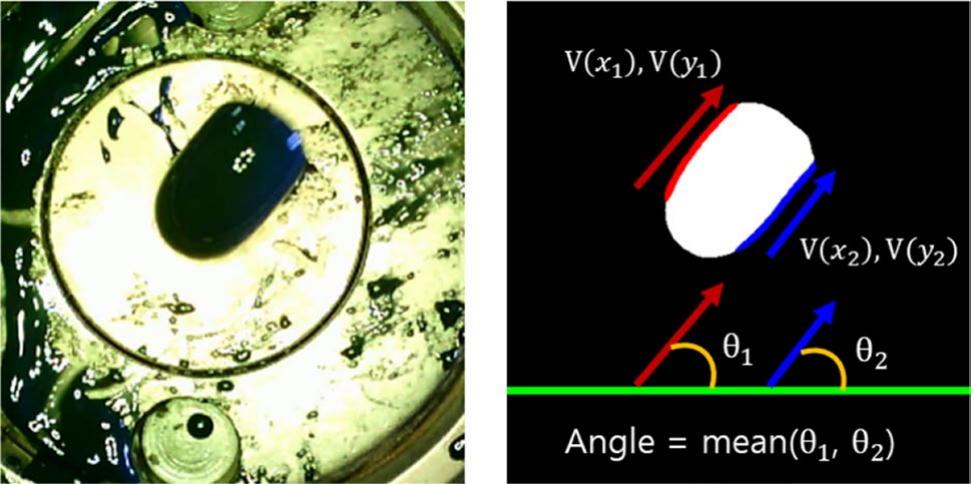
Description of the extrusion angle calculation method.

For the extrusion angle estimation, first, eigenvectors are calculated by using the two-dimensional coordinates of the predicted left and right edge line segments. By using the Karhunen-Loeve transform, the covariance matrix of the input vectors is obtained, and the eigenvectors are sorted according to their eigenvalues^23^. Here, the eigenvector corresponding to the largest eigenvalues indicates the direction in which the variance of the data distribution is the largest. Therefore, the extrusion angle of the resin is calculated by using the eigenvector with the largest eigenvalue. The angle calculation is shown in Eqs. (3) and (4).

3 $$\begin{aligned}{} & {} \theta _{i}(V_{y_{i}},V_{x_{i}})=tan^{-1}({V_{y_{i}},V_{x_{i}}}) \end{aligned}$$

4 $$\begin{aligned}{} & {} {\bar{\theta }}=\frac{1}{n}\sum _{n}^{i=1}\theta _{i}, \end{aligned}$$

where $V_{x_{i}}$ and $V_{y_{i}}$ are the *x*-axis and *y*-axis components of the *i*-th eigenvector. The extrusion angle ${\bar{\theta }}$ is calculated as the average value of each line segment angle calculated using the arctangent function. In this study, the value of n in the Eq. (4) is fixed at 2 because the left and right edge lines are used. The process of calculating the angle is schematized as shown in Fig. 13. By using the calculated angle, the final resin angle is derived by determining the position of the resin among the four quadrants relative to the center line.

### Data acquisition and augmentation

For the extrusion process, a Davis standard 25.4 mm microextrusion system was used. ARKEMA’s PEBAX 7233 was used as the material for the resin extrusion, and the process conditions were set to a temperature of 230 degrees Celsius at the discharge portion and 125 bar as the discharge pressure. These setting conditions are chosen on standardized conditions required for optimal production with the chosen resin. The inner diameter of the die and the outer diameter of the tip used were 5.1 cm and 2.85 cm, respectively.

For the training of the segmentation model, the RGB camera system was installed 15 cm away from the front and side of the discharge part and data was acquired by arbitrarily adjusting the extrusion angle in the frontal images, as shown in Fig. 6. The data set consists of an RGB channel image with 1280- by 960-pixel resolution and a corresponding region mask image. The amount of data for the front and side are 343 and 284, respectively. Each dataset was divided in a ratio of 6:2:2 for training, validation, and testing. During data acquisition, the extruded resin falls when discharged over a certain period due to the influence of gravity. Therefore, in order to estimate the extrusion angle, it is necessary to estimate the angle from the image acquired at the beginning of the extrusion.

**Figure 14:**
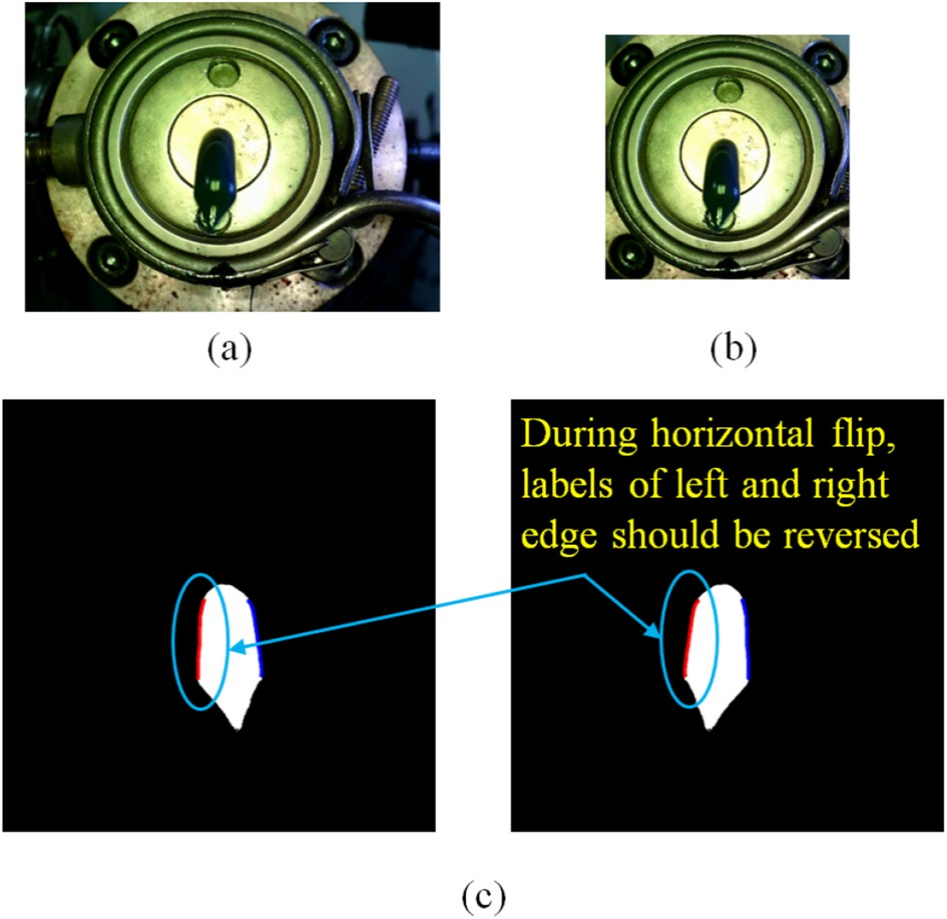
Data augmentation. (**a**) The original 1280 by 960 image, (**b**) cropped image, and (**c**) the horizontally-flipped mask of the cropped image.

For the data augmentation, an image crop and horizontal flip were used. As shown in Fig. 14a and b, an image was cropped to a size of 860 by 860 pixels to reduce the information of the relatively large background area in the image. Since the labels should be different depending on the location of the left and right edges, the left and right labels were reversed during the horizontal flip, as shown in Fig. 14c.

### Training details

For the training, the maximum number of epochs was set to 150. The step learning rate scheduler was applied with an initial learning rate of 0.0001. The learning rate decreases sequentially according to the set number of iterations, which are given as 0.00005, 0.000025, and 0.0000125 for 2,500, 5,000, and 7,500 iterations, respectively. The batch size and dropout rate were set to 3 and 0.5, respectively. Also, Adam was used as an optimizer, and the momentum and weight decay values of the optimizer were set to 0.99 and 0.005, respectively^24^. During training, all networks used pretrained weights from the COCO dataset. The library used for training is torch 1.13.1 based on the CUDA 11.6 version. Also, an RTX 3090 24GB GPU was used for parallel processing.

### Model evaluation

As the presented extrusion angle estimation is based on the segmentation, the accuracy of the segmentation affects the accuracy of the predicted extrusion angle. Because the actual value of the extrusion angle is obtained by applying principal component analysis on the mask image, if the segmentation result is the same as the mask image, the extrusion angle is the same as the actual value. The accuracy of the segmentation is evaluated by using the mean intersection over union (mIoU) which is a commonly used accuracy measure of a segmentation model^22^.

5 $$\begin{aligned} mIoU=\frac{1}{n_{c}}\sum _{i}^{}n_{ii}/\left(t_{i}+\sum _{j}^{}n_{ji}-n_{ii}\right), \end{aligned}$$

where $n_{c}$, $n_{ii}$, $t_{i}$, and $n_{ji}$ are the number of classes, the number of correctly classified pixels—true positive, the total number of pixels in class i, and the number of pixels wrongly not classified—false positive, respectively.

The accuracy of the angle is calculated by using the mean absolute error(MAE) between the predicted and actual angle value in the test samples.

6 $$\begin{aligned} MAE=\frac{1}{N}\sum _{i=1}^{N}abs\left(y_{i}-\hat{y_{i}}\right), \end{aligned}$$

where $y_{i}$, $\hat{y_{i}}$, *N* are the predicted, actual extrusion angle, and the number of test samples, respectively. In order to consider randomness of data shuffling and dropout, the average of value of each accuracy indicators obtained by performing three times experiment is used.

Because the presented method is intended to be installed in a facility with limited computing resources, the calculation time $t_{calc}$ was also measured.

## Results

To verify the validity of the present extrusion resin angle estimation algorithm, an image acquisition device was installed in the extrusion equipment to collect data. By using the collected data, the accuracy of the segmentation model, the accuracy of angle calculation, and the calculation speed were evaluated.

### Experiment results

The accuracy and speed of the ASPP-based segmentation used in this study are greatly affected by the type of backbone network. Therefore, the accuracy and speed according to the representative backbone networks, resnet50, resnet101, and mobilenet are compared.

**Figure 15:**
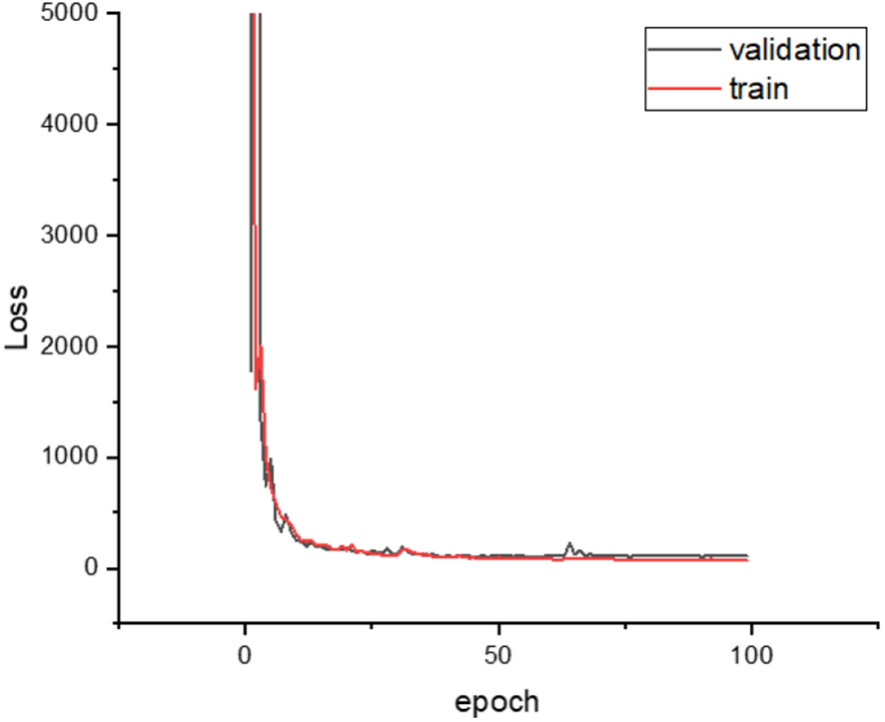
Training and validation loss for the training of the frontal image segmentation model.

First, to check the overfitting phenomenon that may occur during the training process, the training and validation loss are recorded, as shown in Fig. 15. As shown in the figure, it was confirmed that overfitting did not occur because, in the graph, an increase in the validation loss did not occur.

**Figure 16:**
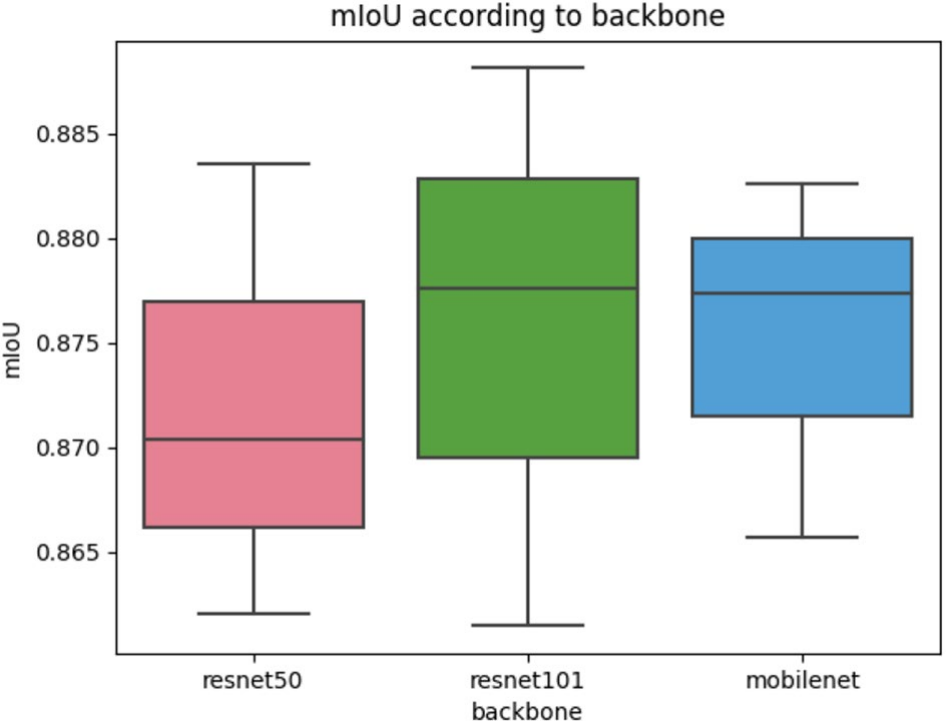
Box plot of the mIoU according to the backbone network.

**Figure 17:**
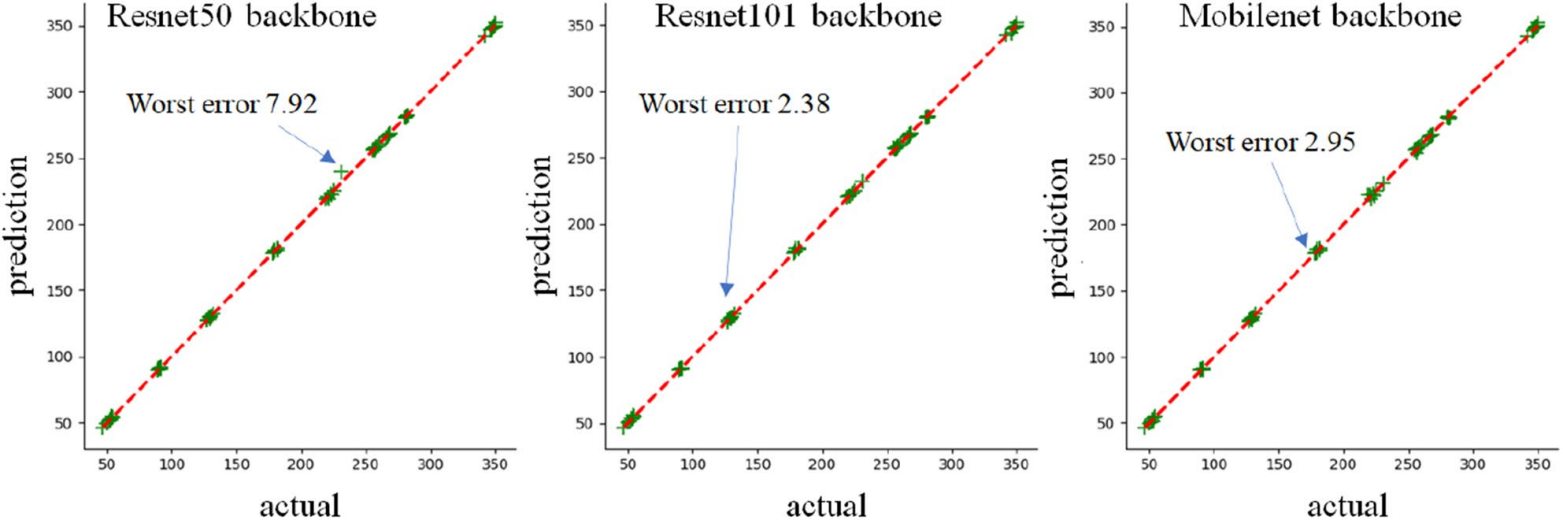
Actual versus prediction plot for the front image according to the backbone network.

Table 1 shows the accuracy results obtained by performing the experiment three times for each backbone network. The mIoU best and MAE best in Table 1 mean the result with the best value of MAE and mIoU among three experiments, respectively. A box plot of the mIoU according to the backbone network is presented in Fig. 16. Also, Table 2 represents the class-specific accuracy of the model that showed the best accuracy for each backbone network. As shown in Table 1, resnet101 showed the best accuracy. Because resnet101 has a relatively deep layer compared to the backbone networks of the other comparator networks, it had the best accuracy. Mobilenet had the worst accuracy but showed better results than the other networks in terms of operation speed. For the frontal image, the calculation time is almost 18 frames per second (fps) with the best average mIoU when the resnet101 backbone network is used. Because 18 fps was judged to be a sufficient speed to infer the angle from the frontal image, resnet101 was determined to be as the best choice for the backbone network. Also, as shown in Table 1, the average MAE is 0.5968 for the front image, which is sufficient for the quantification of the extrusion angle. The actual versus prediction plot of the extrusion angle according to the backbone network is shown in Fig. 17. When resnet50, resnet101, and mobilenet were used as the backbone networks, the highest error values according to the network were 7.92, 2.38, and 2.95, respectively. As shown in Table 2, in the case of class 1 and class 2 representing resin and background areas, they occupy a relatively large area and show a high IoU value, but the IoU of the edge segment area occupying a relatively small area is low. Therefore, in order to increase the accuracy, it is important to accurately classify the edge area.

**Table 1:** Performance of resin segmentation according to the backbone networks.

	Backbone	mIoU best	mIoU average	MAE best	MAE average	Inference time average
Front	resnet50	0.8776	0.8751	0.5875	0.6602	0.0478
	resnet101	0.8881	0.8848	0.5336	0.5968	0.0546
	mobilenet	0.8657	0.8631	0.7176	0.7487	0.0464
Side	resnet50	0.8655	0.8626	1.3748	1.4491	0.0726
	resnet101	0.8673	0.8663	1.2145	1.2976	0.0859
	mobilenet	0.8586	0.8580	1.1942	1.2204	0.0626

**Table 2:** Performance of resin segmentation according to the classes.

	Backbone	IoU best			
		C1	C2	C3	C4
Front	resnet50	0.9995	0.9771	0.7646	0.7691
	resnet101	0.9996	0.9786	0.7850	0.7894
	mobilenet	0.9994	0.9728	0.7478	0.7426
Side	resnet50	0.9993	0.9628	0.7426	0.7574
	resnet101	0.9993	0.9645	0.7497	0.7559
	mobilenet	0.9992	0.9603	0.7355	0.7394

**Figure 18:**
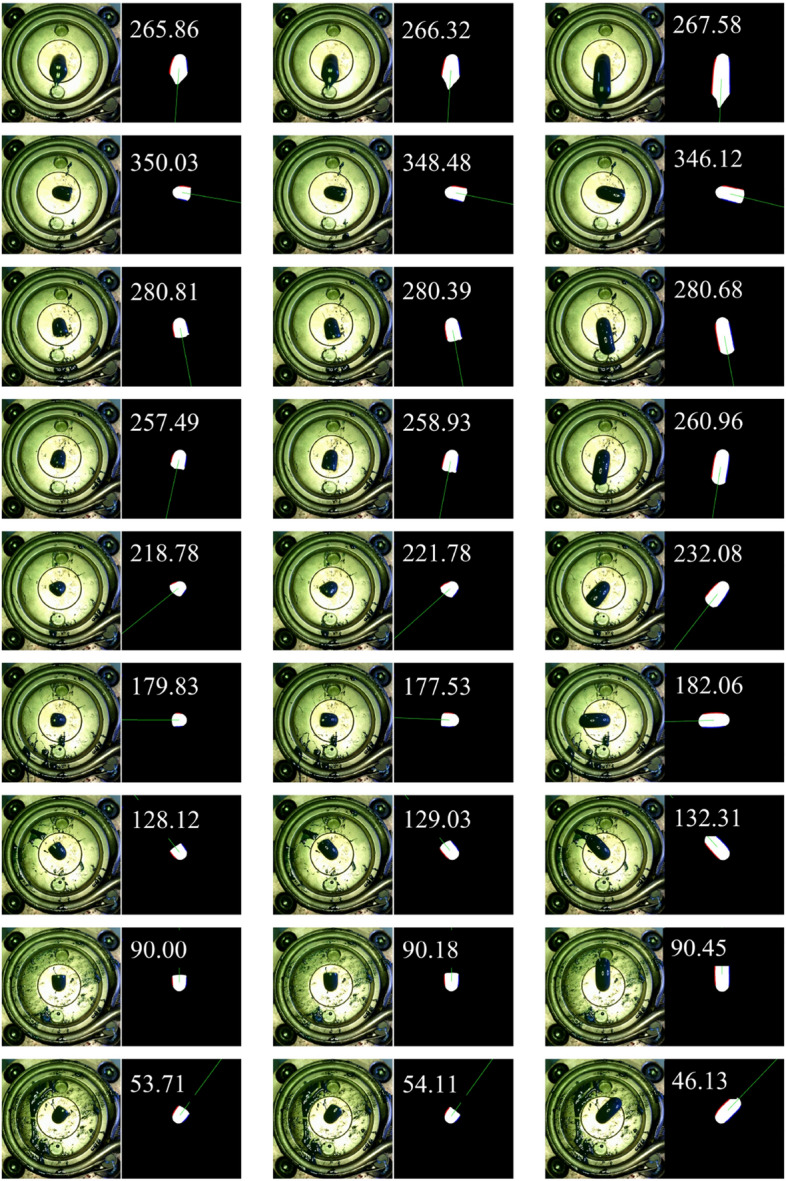
Inference results for the frontal image for each major angle case.

**Figure 19:**
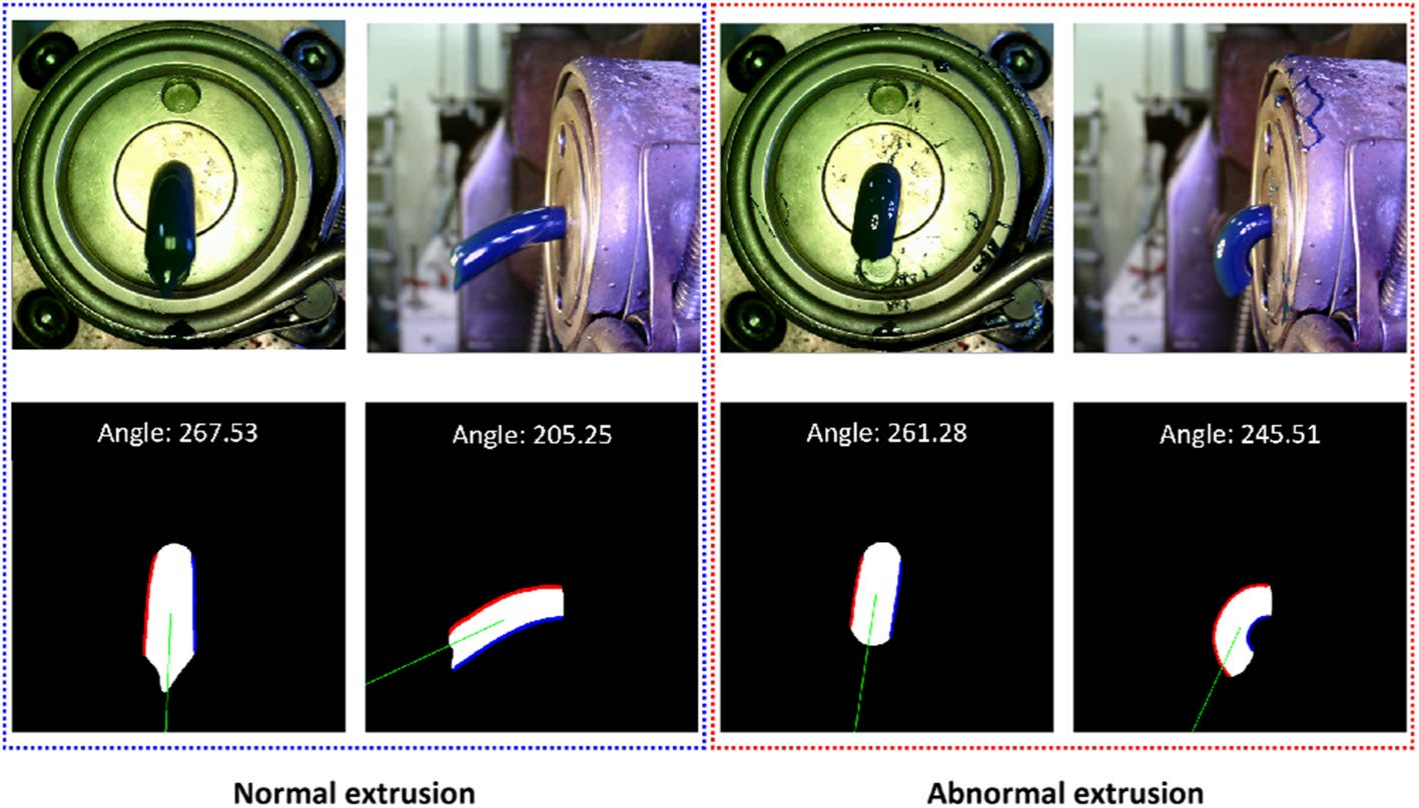
Inference results for the side image to determine top biased extrusion case.

Figure 18 is a visualization of the inference results for the frontal image for each major angle case. As shown in the figure, it was confirmed that quantitative angles can be calculated for major cases that can occur during the extrusion process. The inference result for the side image is shown in Fig. 19. In the case of the side image, it is necessary when the angle value output from the front image is close to 270 degrees. In fact, in most situations, the front discharge angle close to 270 degrees is calculated, but as shown in the figure, the front extrusion angle can be close to 270 degrees even when the resin is biased to the bottom. Therefore, in the case of the side image, it plays a role in determining whether the extrusion is biased toward the bottom. As shown in the figure, if the resin is bent downward and extruded, the initial angle value is over 240 degrees. Therefore, in this situation, it is judged as an abnormal extrusion.

## Conclusion

In this study, a method to quantify the extrusion angle using deep learning was developed to set the initial process conditions for the extrusion process of a catheter tube used in coronary artery surgery. The extrusion process is a traditional manufacturing technique used in various products, and most of the facilities are automated, but, prior to this work, the initial condition setting had to depend on the qualitative judgement of the operator. Due to this setting approach, the produced tubes had uneven quality and resources were wasted in their production. In this study, the extrusion angle was calculated through two procedures. First, the image data obtained from the camera mounted on the front of the discharge part was segmented to extract the edge line area of the resin, and the final resin extrusion angle was calculated using the pixel coordinates of the outer line area. The proposed method was able to confirm that the mIoU, MAE, and inference time were 0.8848, 0.5968, and 0.0546 seconds, respectively, which were applicable to the process. Therefore, through this study, it was confirmed that the deep learning technique can quantify the process output information for setting the initial conditions of the extrusion manufacturing process. In the future, if more data are collected to improve the robustness to disturbances in various process environments and a method for more accurately segmenting the edge line area of the resin is developed, it is expected that process control variables can be automatically controlled.

## Data Availability

The datasets generated and/or analyzed during the current study are not publicly available because they were generated on a commercial product manufacturing line, but are available from the corresponding authors on reasonable request.
